# Visual Deficits and Diagnostic and Therapeutic Strategies for Neurofibromatosis Type 1: Bridging Science and Patient-Centered Care

**DOI:** 10.3390/vision8020031

**Published:** 2024-05-09

**Authors:** Kiyoharu J. Miyagishima, Fengyu Qiao, Steven F. Stasheff, Francisco M. Nadal-Nicolás

**Affiliations:** 1 Retinal Neurophysiology Section, National Eye Institute, National Institutes of Health, Bethesda, MD 20892, USA; kiyoharu.miyagishima@nih.gov (K.J.M.); fengyu.qiao@nih.gov (F.Q.); sfstasheff@childrensnational.org (S.F.S.); 2 Center for Neuroscience and Behavioral Medicine, Gilbert Neurofibromatosis Institute, Children’s National Health System, Washington, DC 20010, USA; 3 Neurology Department, George Washington University School of Medicine, Washington, DC 20037, USA

**Keywords:** NF1, pediatric low-grade glioma, childhood, optic nerve, chiasm, ocular pathologies, animal models, mice, OCT, chemotherapy

## Abstract

Neurofibromatosis type 1 (NF1) is an inherited autosomal dominant disorder primarily affecting children and adolescents characterized by multisystemic clinical manifestations. Mutations in neurofibromin, the protein encoded by the *Nf1* tumor suppressor gene, result in dysregulation of the RAS/MAPK pathway leading to uncontrolled cell growth and migration. Neurofibromin is highly expressed in several cell lineages including melanocytes, glial cells, neurons, and Schwann cells. Individuals with NF1 possess a genetic predisposition to central nervous system neoplasms, particularly gliomas affecting the visual pathway, known as optic pathway gliomas (OPGs). While OPGs are typically asymptomatic and benign, they can induce visual impairment in some patients. This review provides insight into the spectrum and visual outcomes of NF1, current diagnostic techniques and therapeutic interventions, and explores the influence of NF1-OPGS on visual abnormalities. We focus on recent advancements in preclinical animal models to elucidate the underlying mechanisms of NF1 pathology and therapies targeting NF1-OPGs. Overall, our review highlights the involvement of retinal ganglion cell dysfunction and degeneration in NF1 disease, and the need for further research to transform scientific laboratory discoveries to improved patient outcomes.

## 1. Introduction

**Figure 1:**
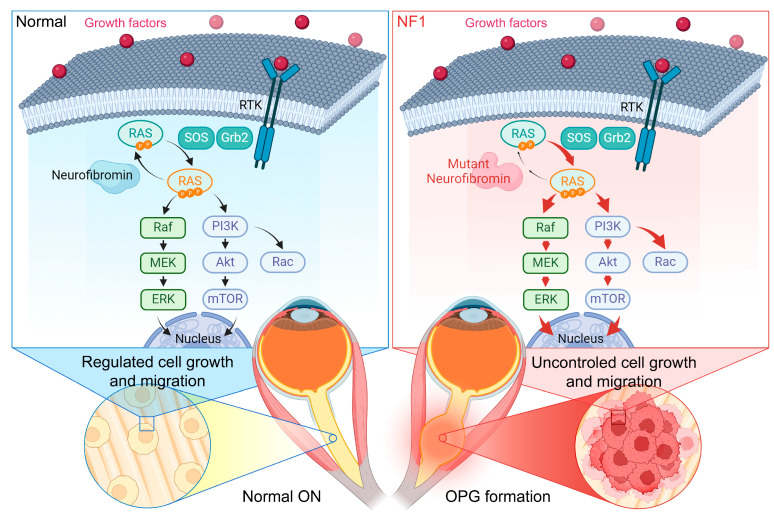
Diagram illustrating the formation of optic pathway gliomas (OPGs) in children with Neurofibromatosis Type 1. Under normal conditions, growth factors stimulate the activation of RAS-GDP to RAS-GTP through son of sevenless (SOS). Neurofibromin (*Nf1* gene) regulates the conversion of RAS-GTP to its inactive form (RAS-GDP), thereby modulating cell growth and migration through the MAPK/ERK and mTOR pathways. In NF1 patients, mutations of neurofibromin significantly reduce its natural activity, resulting in abnormal hyperactivation of the MAPK/ERK and mTOR pathways. Consequently, uncontrolled cell growth and migration lead to the development of gliomas in the optic pathway, potentially affecting vision. Created with BioRender.com.

Neurofibromatosis Type 1 (NF1) is a rare, multifaceted genetic disorder with a complex spectrum of phenotypic clinical manifestations making treatment challenging. The most common feature is café au lait patches on the skin [1]. The prevalence of NF1 is reported to be one in ~3500 individuals [2,3], and although NF1 is an autosomal dominant condition, ~50% of cases occur by *de novo* mutations [4]. NF1 stems from mutations in the *Nf1* tumor suppressor gene, located on chromosome 17q11.2, encoding the neurofibromin protein [5]. Neurofibromin regulates the activity of the RAS-MAPK signaling pathway crucial for cell growth and division [6,7]. In normal conditions, neurofibromin binds RAS to regulate RAF-MEK-ERK activation of the MAPK pathway; however, mutations in the *Nf1* gene result in reduced or absent neurofibromin activity in individuals with NF1, causing uncontrolled cell growth and tumor formation (Figure 1) [8]. Neurological manifestations begin at birth or during early childhood. Individuals are at increased risk of developing tumors of the central nervous system (CNS), including the brain and spinal cord [9]. Clinical manifestations can include benign peripheral nerve sheath tumors, bone deformation, and even curvature of the spine (scoliosis). Scoliosis, which may affect motor abilities, is estimated to be present in ~20% of children with NF1, accounting for approximately ~2% of all pediatric scoliosis cases [10,11]. Features that may also be present include short stature and macrocephaly [12]. Although most NF1 individuals display normal intelligence, learning disabilities are quite common [13,14,15,16].

This review specifically focuses on visual abnormalities associated with NF1. Tumors of the optic nerve associated with NF1, termed optic pathway gliomas (OPGs), are usually benign. OPGs can occur anywhere along the optic pathway, from the optic nerve to the optic chiasm, and they can cause visual disturbances that can be either anatomical (strabismus-eye misalignment) or functional (by decreasing the visual field or visual acuity) [17]. However, some NF1 individuals experience visual deficits that cannot be fully explained by the presence of OPGs. In light of this, there is evidence from animal models suggesting that individuals with NF1 may be at higher risk for retinal ganglion cell (RGC) dysfunction and degeneration [18]. RGCs are a unique neuronal cell type located in the innermost portion of the retina that transmit visual information along their axons (forming the optic nerves) to the brain [19,20]. RGCs are a vital component for visual function and other non-image-forming functions such as circadian photoentrainment and the pupillary reflex [21,22]). Like other neurons in the CNS [23] which lack regenerative capacity [24], RGC injury often leads to cell death and permanent vision loss.

The mechanism underlying RGC degeneration in NF1 involves alterations in genes and signaling pathways that regulate cell growth, differentiation, and survival. Further research is needed to fully understand the relationship between NF1 and RGC degeneration, and to develop effective treatments for this condition. Although some individuals with NF1 have a distinct genotype/phenotype correlation [25,26], heterogeneity in clinical presentation is observed in patients and could be attributed to stochastic events, environmental factors, or modifier genes [27,28,29]. Advances in imaging now allow for non-invasive examination of the retina (optical coherence tomography, OCT) and tumor size and position (magnetic resonance imaging, MRI; magnetic resonance spectroscopy, MRS), offering invaluable “in vivo” measurements. NF1 cases can be examined by infrared fundus autofluorescence (IR-FAF) and OCT to characterize choroidal abnormalities [30] in addition to measurements of retinal nerve fiber layer thickness [31]. Using magnetic resonance imaging (MRI) with contrast enhancement, the optic nerve sheath complex in patients with optic pathway gliomas can be visualized as hypointense on T1-weighted images and hyperintense on T2-weighted images compared to the normal optic nerve [32,33]. Measured parameters include diameter and signal intensity of the optic nerve as well as degree of tortuosity [34]. For diagnostic purposes, imaging and clinical examination is sufficient; however, it is difficult to link these observations to predict vision loss attributed to retinal ganglion cell loss or tumor progression. Ex vivo examination of OPGs is rare as surgical removal is an uncommon practice [35,36]. Thus, the scarcity of enucleated eyes in pediatric patients prevents drawing correlations between RGC quantification, which can only be performed ex vivo, with measurable parameters in patients such as tumor size, type, position, and degree of associated visual impairments. Currently, image analysis and machine learning [37] are being applied to MRIs of optic pathway gliomas to build predictive models that may one day compliment ex vivo methodologies for tumor classification [38,39] or RGC quantification [40,41]. This comprehensive review focuses on neuronal tissues and explores the ocular symptoms associated with NF1, linking current research with advances in diagnostic and therapeutic strategies.

## 2. Phenotypic Manifestations of NF1 Affecting Vision

NF1 ocular manifestations exhibit significant clinical heterogeneity during childhood and adolescence. From overt signs such us Lisch nodules (LNs) to complex ophthalmological complications (such as optic gliomas, plexiform neurofibromas, and congenital glaucoma), there is a full spectrum of pathologies that can influence visual acuity and perceived visual field [42]. LNs, benign tumors with a yellowish-brown dome shape that grow over the iris surface, tend to increase in size and number with age [43,44]. Vision impairment in NF1 patients may stem from various factors, including anatomical causes like proptosis or strabismus originating from intraorbital and periorbital (eye lid and face) plexiform neurofibromas (PLXNs) [17] that may obstruct vision, displace the location of the eye, and interfere with ocular motility; sphenoid wing dysplasia; and the presence of tumors along the optic nerve, OPGs [45,46,47].

**Figure 2:**
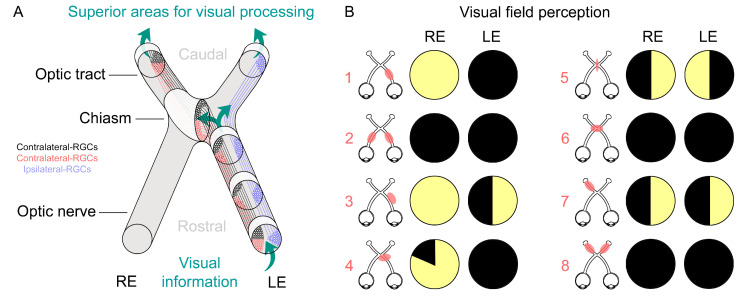
Visual field defects based on the location and size of the axonal damage in the optic pathway. (**A**) Schematic representation of the retinal ganglion cell (RGC) axon projection to superior brain areas for visual processing in the brain. Ipsilaterally (blue) or contralaterally (black and red) projecting RGCs within the optic nerves. (**B**) Depiction of individualized visual field deficits in patients with axonal damage in the optic pathway corresponding to their respective scheme in which red marks represent OPG size and location. Drawings based on concepts presented in [56,57,67].

OPGs are predominantly asymptomatic low-grade gliomas (LGGs), primarily affecting the anterior visual pathway, with 75% occurring in the optic nerve (ON) and optic chiasm. However, they can often involve both ONs, posterior visual pathway segments (optic tract and radiations), and the hypothalamus [48,49]. While mainly pilocytic astrocytomas (grade I), OPGs can also be pilomyxoid astrocytomas and diffuse fibrillary astrocytomas (grade II) [50,51], exhibiting clinical variability. Depending on their behavior, OPGs can be aggressive, leading to visual loss [49,52,53], or regress spontaneously [54]. Symptomatic gliomas typically manifest before age 6, with an average onset of 4.5 years [55]. The location, type, size, and number of OPGs can cause various neurological symptoms affecting visual function and resulting in a range of visual deficits (Figure 2) [56,57]. OPGs may occur unilaterally or bilaterally, be situated anterior to, posterior to, or at the chiasm, centered or asymmetric. The optic tract and even the hypothalamus can also be involved (Figure 2) [53,58]. In extreme cases OPGs can also reach the lateral geniculate nuclei and temporal lobes [59]. Larger tumors and those located closer to the optic nerve are usually associated with more severe visual impairment. Interestingly, patients with gliomas isolated to the optic nerve have better long-term visual outcomes than those with postchiasmatic involvement [60,61]. Additionally, LGGs can affect other brain areas, known as non-OPGs [62]. Although non-OPGs are less frequent than OPGs, they are more frequent in older children/young adults [51] and they can also cause a wide range of neurological symptoms depending on their location, size, and number. These symptoms include headaches, seizures, changes in behavior, cognitive problems, or visual deficits if higher areas of visual processing are involved [63].

Beyond the visual pathway, advancements in multimodal imaging in ophthalmology have revealed microvascular abnormalities in the retinas of NF1 patients [64,65], potentially causing progressive insults of ischemic injury that affect RGC function and viability. Additionally, choroidal abnormalities and hyperpigmented spots have also been observed during ophthalmologic examinations [66]. Although the effects of these choroidal abnormalities on vision are not established yet, the choroid plays a crucial role in maintaining the retinal pigmented epithelium (RPE) and the photoreceptors. Given that photoreceptors are highly metabolically active with high oxygen consumption, choroidal abnormalities impacting oxygen delivery may adversely affect their survival and function and have a profound effect on the patient’s vision. Thus, the diverse ocular manifestations in NF1 individuals highlight the significance of early diagnosis in improving clinical management and enhancing patient outcomes.

Although OPGs are a classic characteristic of NF1 pathology, sporadic OPGs (not associated with NF1) can also form exhibiting distinct genetic hallmarks. In sporadic OPGs, the most common genetic alteration is a duplication of the kinase domain of a gene called B-Raf (BRAF) that leads to MAPK pathway activation, promoting cell survival, growth, and proliferation [68,69,70]. However, other genetic mutations in sporadic OPGs can occur in KRAS, RAF1, FGFR1, PTPN11, and NTRK2 genes [71,72,73,74]. Although NF1 associated OPGs are predominantly found in females, sporadic OPGs occur in both genders with similar frequency [50,75]. Like NF1, sporadic OPGs have a similar age of presentation (4.5 and 5.1, respectively). However, sporadic OPGs are often symptomatic and clinically more severe, with aggressive tumor growth and rapid visual decline compared to OPGs associated with NF1 [75,76,77,78,79]. Notably, studies have determined that sporadic OPGs are predominantly located at the optic chiasm, while OPGs associated with NF1 are generated at the chiasm as well as the optic nerve [76,80,81,82]. Despite their location, sporadic OPGs can lead to a large variety of symptoms such as proptosis, nystagmus, hypothalamic-related endocrine alterations, hydrocephalus, raised intracranial pressure, and vision loss [81,83]. In fact, sporadic OPGs carry a higher risk of vision loss (66–74% in comparison with a 50% risk in NF1-associated OPGs), with visual deficiencies that can appear bilaterally in 25% of cases, and progressive visual loss in 74% of patients regardless of therapy [77,84]. Similar to NF1 OPGs, treatment is necessary only if accompanied by visual impairment. The main treatment is chemotherapy which will be discussed in further detail below.

## 3. Diagnosis and Monitoring Methodologies

The diagnosis of NF1 primarily relies on clinical criteria established by the National Institutes of Health (NIH) in 1987 [85]. These criteria are based on characteristic features such as café au lait spots and neurofibromas. Thus, children meeting clinical criteria for NF1 are expected to undergo regular eye exams to detect asymptomatic OPGs. In fact, several studies have reported a high prevalence of brain tumors in asymptomatic children [44]. Similarly, children presenting with unexplained vision loss, monocular or asymmetric nystagmus, or optic atrophy should be considered for NF1 clinical diagnosis [86]. However, numerous children present significant vision loss prior to receiving treatment, emphasizing the importance of early detection and intervention [87]. Therefore, conducting annual screenings and longitudinal monitoring for signs and symptoms related to OPG throughout childhood is essential for timely clinical decision-making and treatment [88].

In terms of vision, Lisch nodules (LNs) serve as pathognomonic markers of NF1, indicating a potential vision-threatening condition [55]. They appear as small dome-shaped lesions on the iris of the eye and although LNs do not cause visual disturbances, their presence is rare in individuals without NF1. Detecting LNs through a slit lamp examination is a straightforward, noninvasive, and cost-effective method for accurately diagnosing NF1. However, the absence of LNs at earlier stages does not exclude NF1 diagnosis [44]. Importantly, there is no established association between LN presence and the overall clinical severity of NF1 pathology in patients [89,90].

Traditionally, biopsies have been the standard investigation method to confirm NF1 tumor diagnosis. However, they are no longer used routinely, but only exceptionally, to confirm the presence of OPGs [91]. Several imaging techniques offer non-invasive examination of OPGs. Advanced imaging techniques, such as computed tomography (CT) and magnetic resonance imaging (MRI), are ideal to visualize the entire optic nerve or the optic pathway. However, MRI is the preferred method because of superior soft tissue resolution [92,93,94], since OPGs cannot be easily detected on CT scans. Non-contrast MRI provides accurate measurements of tumor size, and contrast-enhanced MRI precisely delineates involvement of other adjacent areas, such as the hypothalamus [94]. MRI can be used to monitor tumor progression in NF1 patients using different approaches, such as volumetric analysis or linear measurements [95]. However, radiographic results to date have provided poor correlation with functional outcomes of patients (visual acuity) in numerous studies [92,93,96].

Interestingly, machine learning algorithms can aid in analyzing the large amount of data generated by these techniques, facilitating initial screening for doctors [97]. Notably, a study reported a correlation between the OPG volume and RGC axon loss [98]. However, systematic MRI screening in children with NF1 has not shown clear benefits yet [99], as early OPG diagnosis and treatment did not improve visual outcomes. Without follow-up examinations, continued optic glioma growth cannot be excluded [8,100]; thus, annual ophthalmologic assessment for changes in the patient’s vision is always recommended.

Optical coherence tomography (OCT) uses reflected near infrared light to produce cross sectional images of retinal tissue structure with a depth of several hundred microns and can help in the diagnosis and evaluation of OPG-associated pathology. In NF1 patients, RGC degeneration can be evaluated by measuring the thickness of the different retinal layers [101]. The ganglion cell complex (GGC) and retinal nerve fiber layer (RNFL [102,103]) thickness provide a quantitative measure of RGC viability. The GCC, comprising the ganglion cell layer and inner plexiform layer, reflects the status of the RGC somas and dendrites, excluding their axons. The RNFL, on the other hand, is primarily composed of RGC axons that converge at the optic nerve head to form the optic nerve. Importantly, the RNFL also includes processes from glial cells (astrocytes, microglia, and Müller cells) that form an intricate interlocking pattern with RGC axons in primate retinas [104,105]. Thus, reactive and infiltrating glial cells in pathological conditions may impact measurements of RNFL thickness, complicating interpretation. Unfortunately, the use of different imaging instrumentation and proprietary software have made it difficult to make direct comparisons among studies [106,107]. In addition, studies in rodents suggest a potential mismatch between RGC loss and RNFL thinning, further complicating interpretation [104,108]. These experiments indicate RGC death precedes axonal atrophy and removal; however, the experimental insult of axotomizing the RGC axons may not recapitulate OPG damage. Despite these caveats, significant thinning of the RNFL or the GCC implies RGC loss, and patients may show substantial visual impairment. Thus, although measurements of RNFL or GCC thickness do not provide a causative link to visual outcomes, they can identify associated anatomical changes to the retina.

Near-infrared imaging (NIR) of the fundus (rear of the eye) generates a 2D image from the amount of reflected light. NIR is able to detect choroidal nodules in NF-1 patients, which appear bright (hyperreflective) and patchy [109]. Although these choroidal nodules do not affect RGCs and their axons directly, they may affect epithelial transport functions of the RPE between the choroid and retina, potentially affecting photoreceptor viability and visual function. Nonetheless, evaluating visual acuity may not identify minor photoreceptor loss, as numerous studies have indicated that a 40–60% loss of cone photoreceptors in the fovea does not have an impact on visual acuity [110,111,112].

Functional tests, particularly visual evoked potentials (VEPs), might play a crucial role in diagnosing and monitoring progression. In response to visual stimulation, VEPs record the generation of electrical impulses from the visual cortex in the brain through electrodes placed in the scalp [31,113]. Reduced amplitudes or delayed responses indicate the magnitude of visual deficits; however, they cannot reveal the nature of the vision loss, and some reports question the correlation between VEP evaluations and vision loss [114,115,116].

A potential future ideal would be molecular and genetic testing to predict the likely phenotype and complications for a person with a specific *Nf1* germline mutation. However, genetic testing is rarely performed due to the extensive heterogeneity in the mutations of the neurofibromin gene and the potential influence of stochastic factors. To date, over 3000 pathological genetic variants of the *Nf1* gene have been identified [117], with less than 20% reported as recurrent [118,119]. Despite the majority of investigations not establishing genotype–phenotype correlations or providing inconclusive results [53], recent studies employing novel screening techniques are beginning to establish correlations [25,120,121,122,123,124,125,126,127,128,129]. The integration of genetic testing in future studies could play a pivotal role in confirming NF1 diagnoses and uncovering genetic variations that may influence ocular phenotypes. Thus, compiling a library of cases has the potential to diagnose and classify new patients earlier, facilitating treatment decision making.

Currently, NF1 gene mutation testing is performed primarily to help confirm or reduce the likelihood of an NF1 diagnosis in cases of clinical uncertainty [86], though genetic testing can also be expanded to include family testing in relatives with uncertain clinical signs or for prenatal testing [6].

Early diagnosis of NF1 patients could be challenging but it is crucial for successful intervention [55,63]. The abovementioned examinations require patient cooperation that can be complicated in preverbal children, especially if they are cognitively compromised. In addition, the natural history of OPGs in NF1 is highly variable; some tumors remain stable, others can regress over time, while others progress, causing significant vision loss. However, these techniques provide crucial information to guide treatment decision, although decisions remain complex [91,130]. Thus, monitoring is an integral part of the management of NF1-associated vision impairment, and examples such as positron emission tomography have proven useful for monitoring OPG progression and response to treatments [131].

## 4. Therapeutic Strategies

Treatment strategies for children with NF1-related visual complications attempt to halt progressive vision loss and promote healthy development. OPG treatment approaches vary depending on specific symptoms, tumor location and size, and extent of visual impairment, and may involve observation, surgical procedures, radiation, chemotherapy, or targeted therapies.

The presence of LNs rarely interfere with vision and typically do not require treatment [44]. However, when OPGs result in the compression of the optic nerve, surgery can reduce symptoms. Surgical procedures are seldom performed to remove pediatric LGGs because they are benign and the likelihood of progression to the chiasm or potential damage to the fibers crossing from the contralateral eye is low [35,36]. Intraorbital and especially intracranial procedures are invasive and carry the risk of vision loss and they can be potentially life-threatening [132], due to bleeding complications [133,134]. Thus, surgical procedures are warranted based on anatomical location and accessibility [135]. Surgery is primarily recommended in cases involving pain, disfiguring proptosis, and/or compression of the surrounding tissues [136]. Although surgery is unlikely to improve vision in patients with orbital OPGs, it may be undertaken for cosmetic purposes [57] or for biopsy if the eye is blind.

Radiotherapy offers an effective treatment for OPGs, but its use is mainly limited to teenagers and those without targeted treatment options due to potential adverse effects, especially in young patients with developing brains [136]. These adverse effects include reduced visual function [137,138], neurocognitive deficits [138,139,140], cerebrovascular abnormalities [141,142], and alterations in endocrine function [143,144] that can persist into adulthood. Novel options include 3D conformal radiation therapy where radiation beams are matched to the volumetric shape of the cancer [145], stereotactic radiosurgery (gamma knife) that focuses the beam to treat smaller targets [146,147], and fractionated stereotactic radiation [148,149] and proton beam radiation [59,150] which minimize damage to healthy surrounding tissue.

Currently, OPGs displaying substantial progression are treated with chemotherapy [47,136,151]. Vincristine and carboplatin are often prescribed as the first line of treatment and have shown reasonable progression-free survival rates at earlier stages [152]. However, carboplatin doses can lead to hypersensitivity and frequency-based adverse effects in some individuals [153]. Alternative combinations, such as cisplatin and etoposide [154] or thioguanine, procarbazine, and lomustine appear to improve event-free survival [155]. However, caution on the use of these alternative drugs in treating NF1 is advised due to the risk of developing secondary leukemia attributed to etoposide [156], and to procarbazine and lomustine [154,157]. Monotherapies with vinblastine [158,159], vinorelbine [160], trametinib [135,161] or temozolomide [162] have also shown efficacy in NF1 patients with low toxicity, except there have been reported cases of secondary leukemia following temozolomide and radiotherapy [163]. Notably, pre-chiasmatic OPGs appear to be more responsive to chemotherapy than gliomas located elsewhere in the optic pathway [164]. However, while chemotherapy often limits tumor growth effectively, very few individuals show visual improvement following treatment [96], particularly patients with late-progressive OPGs [165].

Another set of treatments are targeted therapies. Bevacizumab is an anti-vascular endothelial growth factor (VEGF) monoclonal antibody [166] that reduces vascular permeability and tumor growth. It improves visual symptoms in most cases [167,168] when administrated alone or in combination with irinotecan, a DNA topoisomerase I inhibitor that interrupts DNA replication and cancer growth [169,170,171,172]. However, bevacizumab causes reversible side effects (hypertension, fatigue, joint pain, bleeding, and proteinuria) that can persist after treatment ending, and tumor progression is common after treatment discontinuation [167,171,173].

Novel agents focus on inhibiting the mitogen-activated protein kinase (MAPK) pathways. Selumetinib, a selective MEK1/2 inhibitor, has been shown to maintain or improve visual acuity after oral administration in patients with OPGs [174]. Recent studies show MEK inhibitors such as refametinib, trametinib, and cobimetinib can shrink the volume of most inoperable benign LGGs and malignant plexiform neurofibromas, improving neurocognitive function in NF1 patients [175,176]. In fact, MEK inhibitors have also demonstrated tumor suppression in preclinical mouse models [177]. However, it is important to note that the response of LGGs to MEK inhibition is often variable, and regrowth is frequently observed after discontinuation of therapy [177]. Other promising treatment options include small competitive molecules (vemurafenib and dabrafenib) that prevent bRAF from binding MEK and activating the MAPK pathway [178]. Rapamycin and its derivates, such as everolimus, are selective mTOR blockers. A recent study demonstrated oral administration of everolimus stabilized visual acuity in children with NF1-OPGs with low levels of toxicity [179,180].

In addition, administration of pro-survival factors can preserve RGC survival or stimulate axonal regrowth, as demonstrated by a clinical study in which NF1 patients were given eye drops with murine nerve growth factor for 10 days, and a third of the treated group showed significant improvement in the size of their visual field [181].

Overall, the management of NF1-associated vision impairment requires a multidisciplinary approach, involving close collaboration between ophthalmologists, oncologists, neurologists, geneticists, neurosurgeons, endocrinologists, and pathologists. Early diagnosis is crucial to initiate treatment promptly and prevent irreversible visual decline, while regular monitoring (tumor size, visual function) is essential to ensure optimal outcomes in patients with NF1 and OPGs.

## 5. The Role of Animal Models to Uncover Underlying Mechanisms of NF1 and to Develop Novel Therapies

The scarcity of surgical resection or biopsies from OPGs in NF1 patients underscores the utility of preclinical animal models in providing knowledge about these tumors.

Genetically engineered rodents, particularly mice, are the most widely used and best characterized models of NF1-OPG. Generation of *Nf1* knockout mice from the germline (*Nf1^−/−^)* was unsuccessful as they were lethal and their heterozygous littermates (*Nf1^+/−^)* did not develop astrocytomas despite increased astrocyte proliferation [182,183]. Subsequent studies focused on germline mutations in *Nf1* resulting in varying levels of neurofibromin expression and the development of optic gliomas [184]. Currently, the most successful mouse models are the conditional knockout lines, which allow for inactivation of *Nf1* in specific cell lineages. Utilizing the Cre-lox system enables the generation of NF1-associated tumors in animals without being lethal. In this context, specific deletion of the *Nf1* gene in astrocytes (*GFAP-Cre; Nf1^flox/mut^*) successfully induced formation of OPGs [185,186]. Additional mouse lines have been developed that more closely resemble NF1 human pathology by introducing human GFAP (*hGFAP-Cre; Nf1^flox/mut^*) [187]. This line exhibited complete penetrance of glial hyperplasia and enlarged optic nerves with lesions that in some cases progressed to form optic pathway gliomas [188]. Other mouse models achieve OPG formation by activating the KRAS oncogene in astrocytes of heterozygous *Nf1* mice [189], or inhibiting *Nf1* in neuroglial progenitors (such as BLBP and Oligo2 [190,191]) thereby increasing proliferation of cells with glial lineage and inducing abnormal neuronal differentiation. However, a recent report suggests that in *Nf1*-deficient neuroglial progenitor cells, CNS injury could be sufficient to induce glioma formation, indicating that independent injuries can promote tumor development in susceptible animals [192]. Mouse models have contributed to our understanding of OPG formation and have highlighted mechanisms for mTOR-dependent glioma formation [193], implicating microglia in glioma formation [194,195,196,197], and the presence of glioma-specific stem cells [198,199]. In addition, mouse models are a valuable tool to design novel therapeutic strategies or redefine existing treatments. Animal studies have helped define the therapeutic window to rescue neural progenitors by administration of MEK/ERK inhibitors during early postnatal stages [200]. Interestingly, Jecrois and colleagues reported that either the simultaneous removal of three out of the four alleles from the *Mek1* and *Mek2* genes (as complete elimination of the two alleles of Mek1 and the two alleles of Mek2 proved lethal) or administration of a low-dose MEK inhibitor (PD0325901) through the lactating mother’s milk prevented NF1-OPG formation [201]. A recent report attributed the predominance of OPG formation in girls to higher levels of glial interleukin-1β, which can be suppressed by IL-1β neutralization and leuprolide-mediated estrogen suppression [192,202]. However, it is important to note that the formation of the optic chiasm in mice differs from humans [181] and these tissue-restrictive tumors in mice do not fully replicate the complex pathology of NF1 patients [203]. Additionally, humans have a different proportion of ipsilateral and contralateral projecting RGCs, with ~50% of RGC axons decussating to the contralateral optic tract, while in rodents, only a few RGCs contribute to binocular vision (with ~95–97% of RGCs projecting contralaterally [204,205,206]).

Large animal models of NF1 offer a better anatomical comparison to understand the NF1 pathology in humans. Genetically engineered porcine models, such as the *Nf1^+/R1947X^* minipigs, share major clinical NF1 features. Electron microscopic evaluation of the optic nerves demonstrated significant demyelination and OPG formation that was confirmed by MRI and CT scans [207,208]. Additionally, their comparable eye and optic nerve size to humans can facilitate the development of new imaging approaches for diagnosis and the testing of novel surgical modalities. Their body size, metabolism and lifespan make them an ideal preclinical model for longitudinal studies, pharmacological tests, and drug dose optimization studies [203]. Large animal models may facilitate translational studies, acting as an intermediate between small rodents and humans [180]. Similarly, cases of spontaneous NF-like manifestations in large animals, such us cattle and dogs [203,209,210], are highly valuable because they can provide insights into the natural malignant transformation of some tumors, contrasting with genetically engineered models [211].

From a different perspective, the zebrafish model can facilitate large-scale experiments for treatment screening, as generating transgenic lines is cost-effective since they are easy to handle and have a high fertility rate [212,213]. The drosophila model can also contribute to large-scale testing; however, these studies typically focus on peripheral nerves, social abilities, and development [214,215]. In addition to animal models, cell culture methods also enable high-throughput screening, which could be particularly valuable when using cells developed from patients to generate compact LGGs [216].

An integrative approach requires collaboration between clinicians and researchers. This collaboration not only enriches our understanding of the disease but also bridges the gap between clinical observations and laboratory advancements. Furthermore, neuroscientists are exploring novel strategies to promote RGC survival and regeneration, including neuroprotective drugs [217], gene therapy [218], or stem cell transplantation [219,220]. These approaches hold promise for improving the vision of NF1 patients; however, most are still in the experimental stage, requiring further research to determine their safety and efficacy.

## 6. Concluding Remarks

The study and treatment of NF1 pose significant challenges owing to its variable clinical presentation and biological complexity. Clinically, NF1 exhibits remarkable heterogeneity, manifesting diverse symptoms and complications, with variability in penetrance and unpredictable progression of associated OPGs and retinal abnormalities. The major challenge in understanding NF1 pathophysiology arises from the extensive genetic and phenotypic variability, coupled with the absence of clear associations with visual deficits.

Further complicating NF1-related retinal research is the lack of well-defined biomarkers that can differentiate between asymptomatic and symptomatic OPGs that lead to vision loss. Unlike systemic aspects of NF1 that are accessible and more easily monitored, the intricate structure and function of the retina and brain demand sophisticated imaging techniques and functional assessment, which may not translate readily into easily measurable biomarkers. Despite these challenges, progress in genetic research, development of new imaging technologies, and collaborative efforts are gradually enhancing our understanding of NF1.

Ethical considerations introduce additional complexity, particularly in dealing with pediatric populations who cannot consent to clinical investigations for themselves. The hereditary nature of NF1 requires careful navigation of informed consent from parents/guardians and privacy concerns. This highlights the need for an ethical framework that respects the rights and well-being of patients, making the assembly of large patient cohorts difficult, and limiting robust data analysis. This emphasizes the importance of collaborative efforts across research centers to consolidate data and share insights.

While animal models have been developed to better understand the mechanism of NF1 pathology and to design new therapeutic approaches, replicating the full spectrum of abnormalities to the retina, optic nerve, and visual pathways observed in humans remains a significant challenge. While mouse lines can mimic certain features of the NF1 pathology, species-specific differences in ocular/brain anatomy and physiology may limit translation to the human condition. Large animal models such as minipigs exhibit a closer resemblance to human anatomy and physiology. Newer models are being developed to better replicate human NF1 pathology, and to shed light on NF1-related visual impairment.

Integration of clinical observations, advanced imaging technologies, and molecular analyses requires collaboration among diverse fields, including ophthalmology, genetics, neurology, and basic science research. Moreover, understanding the interplay between genetic and environmental factors in the development and progression of retinal abnormalities associated with NF1 is crucial. Untangling these interactions is essential to understanding the underlying causes of many of the NF1-related retinal manifestations. Through this integrative review, we summarize the ocular facets of this inherited disorder and encourage synergistic collaboration to discover therapeutic interventions to mitigate visual loss in NF1-affected individuals.

## Data Availability

Not applicable.
